# Efficacy of post-operative radiation in a prostatectomy cohort adjusted for clinical and genomic risk

**DOI:** 10.1038/pcan.2016.15

**Published:** 2016-05-03

**Authors:** A E Ross, R B Den, K Yousefi, B J Trock, J Tosoian, E Davicioni, D J S Thompson, V Choeurng, Z Haddad, P T Tran, E J Trabulsi, L G Gomella, C D Lallas, F Abdollah, F Y Feng, E A Klein, A P Dicker, S J Freedland, R J Karnes, E M Schaeffer

**Affiliations:** 1 James Buchanan Brady Urological Institute, Johns Hopkins Hospital, Baltimore, MD, USA; 2 Sidney Kimmel Medical College, Thomas Jefferson University, Philadelphia, PA, USA; 3 GenomeDx Biosciences, Vancouver, BC, Canada; 4 EMMES Canada, Burnaby, BC, Canada; 5 Department of Radiation Oncology, Johns Hopkins Hospital, Baltimore, MD, USA; 6 Vattikuti Urology Institute, Henry Ford Hospital, Detroit, MI, USA; 7 Department of Radiation Oncology, University of Michigan, Ann Arbor, MI, USA; 8 Glickman Urological and Kidney Institute, Cleveland Clinic, Cleveland, OH, USA; 9 Department of Surgery, Division of Urology, Center for Integrated Research on Cancer and Lifestyle, Samuel Oschin Comprehensive Cancer Center, Cedars-Sinai Medical Center, Los Angeles, CA, USA; 10 Surgery section, Durham Veteran Affairs Medical Center, Durham, NC, USA; 11 Department of Urology, Mayo Clinic, Rochester, MN, USA; 12 Department of Urology, Northwestern University, Evanston, IL, USA

## Abstract

**Background:**

To date, there have been no published trials examining the impact of salvage radiation therapy (SRT) in the post-operative setting for prostate cancer (PCa). We conducted a retrospective, comparative study of post-operative radiation following radical prostatectomy (RP) for men with pT3 disease or positive margins (adverse pathological features, APF).

**Methods:**

422 PCa men treated at four institutions with RP and having APF were analyzed with a primary end point of metastasis. Adjuvant radiation treatment (ART, *n*=111), minimal residual disease (MRD) SRT (*n*=70) and SRT (*n*=83) were defined by PSA levels of <0.2, 0.2–0.49 and ⩾0.5 ng ml^−1^, respectively, before radiation therapy (RT) initiation. Remaining 157 men who did not receive additional therapy before metastasis formed the no RT arm. Clinical–genomic risk was assessed by Cancer of the Prostate Risk Assessment Post-Surgical (CAPRA-S) and Decipher. Cox regression was used to evaluate the impact of treatment on outcome.

**Results:**

During the study follow-up, 37 men developed metastasis with a median follow-up of 8 years. Both CAPRA-S and Decipher had independent predictive value on multivariable analysis for metastasis (*P*<0.05). Adjusting for clinical–genomic risk, SRT and no RT had hazard ratios of 4.31 (95% confidence interval, 1.20–15.47) and 5.42 (95% confidence interval, 1.59–18.44) for metastasis compared with ART, respectively. No significant difference was observed between MRD-SRT and ART (*P*=0.28). Men with low-to-intermediate CAPRA-S and low Decipher value have a low rate of metastatic events regardless of treatment selection. In contrast, men with high CAPRA-S and Decipher benefit from ART, however the cumulative incidence of metastasis remains high.

**Conclusions:**

The decision as to the timing and need for additional local therapy following RP is nuanced and requires providers and patients to balance risks of morbidity with improved oncological outcomes. Post-RP treatment can be safely avoided for men who are low risk by clinical–genomic risk, whereas those at high risk should favor enrollment in clinical trials.

## Introduction

Aggressive treatment approaches that have not yet been shown to improve overall survival are controversial within oncology, particularly in the management of men with prostate cancer (PCa).^1, 2^ Three randomized clinical trials in men with non-metastatic PCa following surgical resection showed that treatment with adjuvant radiation therapy (ART) as compared with observation resulted in lower rates of biochemical recurrence, yet had conflicting impact on metastasis-free and overall survival.^3, 4, 5^ Given the disparate findings, there has been an increase in utilization of salvage radiation therapy (SRT)^6^ with concomitant decrease in ART, despite no randomized prospective trial evidence supporting this clinical decision. Although multiple randomized clinical trials are ongoing,^7, 8^ results are not expected for several years. Thus, patients and physicians are left with much consternation and doubt as they attempt to balance potential toxicities from overtreatment with the possibility of missing a window for cure.

Currently, in the immediate post-operative period risk models exist, which can predict an individual patient's risk of metastatic progression. Among these are clinical derived risk models, such as Cancer of the Prostate Risk Assessment Post-Surgical (CAPRA-S), which combines clinical and pathological data via the use of a scoring system.^9, 10^ CAPRA-S has been externally validated for prediction of biochemical recurrence, disease progression and PCa-specific mortality.^11, 12^ More recently, tissue-based genomic testing in the form of Decipher has been developed and validated to predict metastasis-free survival.^13, 14^ Decipher has been examined in multiple cohorts and post-prostatectomy settings and has been found to be an independent predictor of metastasis among men followed expectantly and those receiving post-operative ART and SRT.^15, 16, 17^ CAPRA-S and Decipher have also been shown to potentially help in the selection of men for ART as opposed to SRT, but these studies have lacked an untreated post-prostatectomy cohort and were subject to bias. Here, using a multi-institutional database, we evaluate the combination of clinico-pathological and genomic risk in the context of post-operative therapeutic choices including adjuvant and salvage therapy as well as expectant management of disease with adverse pathological features.

## Materials and methods

### Patient cohort

**Figure 1:**
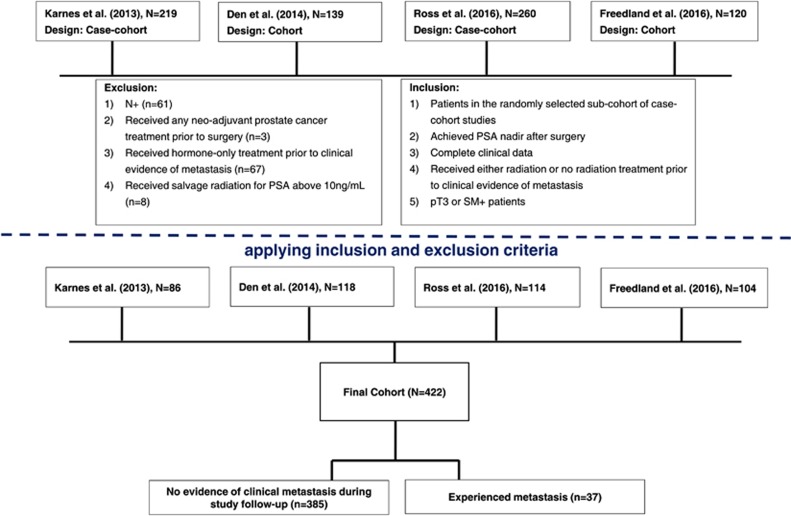
Study diagram.

Multiple prior studies have demonstrated that the presence of adverse pathological features defined as positive surgical margins, extracapsular extension or seminal vesicle invasion portend for higher rates of biochemical recurrence, development of metastases and death from PCa.^4^ A total of 422 patients with PCa treated with radical prostatectomy (RP) between 1990 and 2010 who had adverse pathological features, and no lymph node metastasis were identified from four academic institutions; Mayo Clinic (*n*=86); Durham Veterans Affairs (*n*=104); Johns Hopkins Medical Institution (*n*=114); and Thomas Jefferson University (*n*=118); see Figure 1 for schematic representation. Patient tumors were deposited into the GenomeDx PCa genomic resource information database; institutional review boards at the participating institutions approved the research protocol under which the data were collected.

All patients reached an undetectable PSA following surgery. Patients received either no post-operative treatment before development of metastasis or were treated with either ART or SRT using three-dimensional conformal radiation therapy or intensity modulated radiation therapy to a median dose of 66.6 Gy using conventional fractionation. There was no statistical difference in the use of intensity modulated radiation therapy, pelvic fields or concurrent androgen deprivation therapy between men who underwent ART or SRT.^18, 19^

The primary end point for the analysis was incidence of clinical metastasis (regional or distant) documented radiographically on computed tomography or bone scan. ART, minimal residual disease SRT (MRD-SRT) and SRT were defined by initiation of therapy at PSA levels of <0.2, 0.2–0.49 and ⩾0.5 ng ml^−1^, respectively. Patients who did not receive additional therapy (RT or ADT) before metastatic onset were considered as the control group defined as ‘no RT'. Patients who received SRT with a pre-raditation therapy (RT) PSA >10 ng ml^−1^ were excluded from the analysis (*n*=8). In addition, eight patients received ADT after RT and none of the patients received any other systemic agents (other than ADT) before metastatic onset.

### Specimen collection and handling

Specimen selection and processing has been described previously.^20, 21^ Following microarray quality control using the Affymetrix Power Tools packages,^22^ probeset summarization and normalization was performed utilizing the single-channel array normalization algorithm.^23^ None of these samples were used in the development of the Decipher genomic classifier.^24^

### Calculation of clinical and genomic risk of metastasis

Clinical risk of metastatic progression was calculated with CAPRA-S score using six clinico-pathological variables as described previously.^9^ Genomic risk of metastatic progression was calculated with the Decipher test. In brief, expression values for the 22 pre-specified biomarkers that constitute Decipher were extracted from the normalized data matrix and entered into the locked random forest algorithm with tuning and weighting parameters defined as reported previously.^20^ The Decipher read-out is a continuous risk score between 0 and 1, with higher scores indicating a greater probability of metastasis.^24^ Decipher scores were rounded to two significant digits.

### Statistical analysis

To compare clinico-pathological variables across treatment groups, Fisher's exact test and the analysis of variance F-test were used for categorical and continuous variables, respectively. In time to event analyses, event times were defined as the time from RP to metastasis. Cumulative incidence curves were constructed using Fine–Gray competing risks analysis to estimate the risk of metastasis over time.^25^ Cox univariable and multivariable proportional hazards model was used to evaluate the impact of treatment on outcome after adjustment for CAPRA-S and Decipher. Predicted risk curves were based on a Kaplan–Meier estimate of the baseline risk. The results of the multivariable model were confirmed by competing risks regression and Firth's penalized likelihood method, with no substantive change in hazard ratios or *P*-values.^26, 27^ Tests for treatment interaction with CAPRA-S and Decipher were also performed. In a sensitivity analysis, a multivariable Cox proportional hazards model was fitted with MRD-SRT and SRT treated as time-dependent covariates. All statistical tests were two-sided and analyses were performed in R v3.1 (R Foundation, Vienna, Austria).

## Results

Out of the 422 men with adverse pathological features, 37 developed metastasis. Median follow-up among those who did not develop metastasis was 8 years (interquartile range (IQR), 5–11). Clinical and pathological variables for each treatment group are summarized in Table 1. Extraprostatic extension and positive surgical margins rates were significantly different among the treatment groups (both *P*<0.001) with the no RT group having the lowest positive surgical margin rate and highest rate of extraprostatic extension. Median PSA at the time of MRD-SRT was 0.30 ng ml^−1^ (IQR, 0.25–0.40) and 1.00 ng ml^−1^ (IQR, 0.65–2.20) at the time of SRT. During study follow-up, 3 (3%), 4 (6%), 11 (13%) and 19 (12%) patients developed metastases in the ART, MRD-SRT, SRT and no RT groups, respectively. Median follow-up among censored patients was 7 (IQR, 5–10), 8 (IQR, 5–11), 8 (IQR, 5–12) and 8 (IQR, 5–11) years for ART, MRD-SRT, SRT and no RT groups, respectively.

Distribution of CAPRA-S and Decipher risk scores are depicted in Supplementary Figure S1. On the basis of previously defined CAPRA-S risk categories,^15^ 6, 58 and 36% of men were classified as low (0–2), intermediate (3–5) and high risk (6-12), respectively and the cumulative incidence of metastasis at 10 years post RP was 11.3, 3.3 and 21.4%, respectively. In contrast, Decipher score classified 57, 27 and 16% as low (<0.45), intermediate (0.45–0.60) and high risk (>0.60), respectively. Cumulative incidence of metastasis at 10 years post RP was 6.8, 10.3 and 21.9% for these risk groups.

On multivariable analysis, both CAPRA-S and Decipher scores were independent predictors of metastasis (Table 2 and Supplementary Table S1). SRT and no RT had a hazard ratio of 4.31 (95% confidence interval, 1.20–15.47) and 5.42 (95% confidence interval, 1.59–18.44) compared with ART, respectively, when adjusting for CAPRA-S and Decipher. In contrast, no statistical difference was seen when comparing MRD-SRT to ART (*P*=0.28). Adjusting for concurrent ADT with RT did not significantly alter the results (Supplementary Table S2). Results of the multivariable model remained similar when MRD-SRT and SRT were treated as time-dependent covariates or when patients (*n*=8) with ADT after RT were excluded from the analysis (Supplementary Tables S3 and S4). Because there was no evidence that treatment effect was dependent on baseline clinical or genomic risk (*P*=0.16 for CAPRA-S and *P*=0.39 for Decipher), subsequent survival models did not include an interaction term.

**Figure 2:**
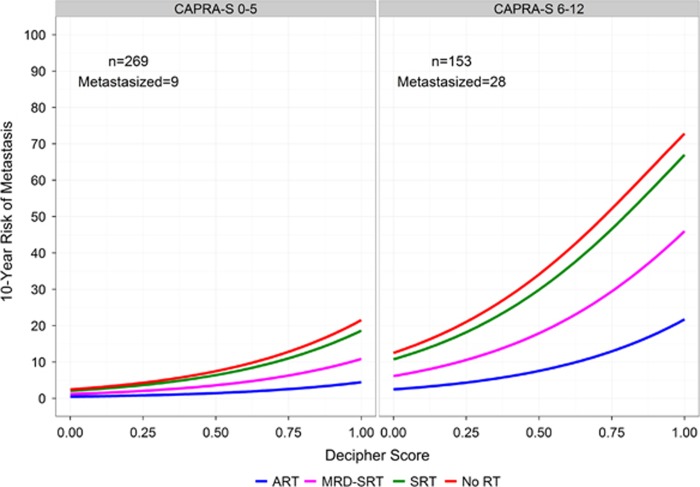
Prediction curves of metastasis for treatment groups at 10 years adjusted by categorical Cancer of the Prostate Risk Assessment Post-Surgical (CAPRA-S) and Decipher score. ART, adjuvant radiation therapy; MRD, minimal residual disease; SRT, salvage radiation therapy.

Prediction curves for 10-year risk of metastasis based on these models are presented in Supplementary Figure S2. For both risk models (CAPRA-S or Decipher), 10-year risk of metastasis increased consistently with rising scores. Among men at low or intermediate Decipher risk, there was a wider separation of metastatic outcomes based on treatment group when compared with men with low or intermediate CAPRA-S scores. Predicted risk of metastasis at 10 years post RP increased consistently with rising CAPRA-S scores, from 0 to 22% for ART, from 1 to 45% for MRD-SRT, from 2 to 65% for SRT and 2 to 70% for no RT group. Similarly, predicted risk of metastasis increased consistently with rising Decipher scores, from 1 to 21% for ART, from 2 to 33% for MRD-SRT, from 4 to 64% for SRT and 4 to 65% for the no RT group. Group level 10-year risk of metastasis for CAPRA-S score, and Decipher score are provided in Supplementary Table S5. As both CAPRA-S and Decipher scores had independent prognostic ability for metastasis, we constructed prediction curves for 10-year risk of metastasis taking both factors into account (Figure 2). In a sensitivity analysis, similar results were observed when ART 10-year risk of metastasis was adjusted using the formula described by King (Supplementary Figure S3).^28^

## Discussion

Despite its high incidence, the optimal management for PCa remains contentious. In the post-prostatectomy setting, a uniform strategy is inadequate and can result in simultaneous over- and under-treatment resulting in unnecessary toxicity and burden to the health-care system as well as missed opportunities for cure. The imprecise identification of patients at highest risk of metastatic disease and death from PCa highlights the need for additional risk stratification beyond the clinical features. Herein we also incorporate both clinical and genomic information providing improved assessment of metastatic risk in the context of post-operative radiation.

Our group has previously reported decreased incidence of metastasis in men who underwent ART (6%) as compared with SRT (23%) in the intermediate to high genomic-risk groups.^16^ However, advocates of salvage radiation have always postulated that RT initiated at low PSA values (we have coined this MDR-SRT) is as effective as ART. In this work, with a much larger population of men, we explore differences in outcomes of salvage RT based on PSA values. Multivariable analysis revealed SRT (PSA ⩾0.5 ng ml^−1^) and no RT were associated with an approximate fivefold increased rates of metastasis when compared with ART or SRT administered in the setting of MRD (PSA 0.2–0.49 ng ml^−1^). These findings are consistent with the approximate fourfold risk observed by Den *et al.*^16^, when initiation of RT was dichotomized around a PSA threshold of 0.2 ng ml^−1^ and support a beneficial effect of earlier application of secondary local treatment in attaining longer term oncological control as was observed in clinical trials.^29^ There appeared to be minimal utility associated with SRT instituted at higher PSA levels (⩾0.5 ng ml^−1^), supporting previous evidence that the window of curability following biochemical recurrence remains small, and that radiation therapy has maximal oncological benefit when delivered at low PSAs.^30^ Importantly, the data does not suggest that SRT be omitted in men with higher PSA levels but rather that radiation therapy alone in these men is unlikely to be curative and strong consideration should be given to the addition of systemic therapy. Furthermore, we found that PSA level following prostatectomy as opposed to timing of radiation was the major determinant of response (with no significant difference in the timing of radiation and an earlier median time to radiation among the SRT patients when compared with MRD-SRT patients). Of note, we found no significant difference in metastases risk between ART and MRD-SRT. However, as the hazard ratio of MRD-SRT was >2 with wide confidence intervals, it is unclear whether MRD-SRT does indeed provide similar efficacy as ART or whether our study was underpowered to detect a benefit of ART. Ultimately, more studies are needed to address this point.

There are major clinical ramifications to this study as there is increased utilization of prostatectomy amongst men with intermediate and high-risk disease—as these men are more likely to have adverse pathological features and subsequent biochemical recurrence.^10^ Given the multiple competing factors influencing patient decision-making, more personalized guidance is needed for this population. Genomic risk stratification assays guide treatment decisions and improve outcomes.^31, 32^ This study provides men and their physicians with risk-estimates for metastatic development, which empowers patients to tailor therapy to meet their values and desires through using an individualized threshold of risk (Figure 2).

In addition to its retrospective design, this study has limitations that merit discussion. First, treatment approach was not randomly assigned and thus varied based on provider biases, institutional biases and patient preferences. Although we adjusted for risk among the cohorts, it is important to note that some men in the ART cohort would be expected to have been cured with surgery alone while all men receiving SRT by definition failed initial attempts for disease control and this bias, although accounted for, remains a limitation for comparisons of ART to SRT. Further, while patients receiving observation only were imaged at the time of biochemical recurrence and then yearly thereafter until the time of metastasis, those patients receiving radiation therapy did not have systematic yearly imaging, often with serial staging performed only after PSA rise or once they were symptomatic. Thus, ascertainment bias may have accounted for some of the differences between groups. In addition, while greater than in previous studies, the sample size and number of observed events limited the study power. Further, data regarding men with PSAs detected at an ultrasensitive range were not available, and thus no conclusions can be made in that regard. This study had several distinctive strengths, including long median follow-up, a no RT ‘natural history' group in addition to well defined adjuvant and salvage therapy groups and use of a validated genomic and clinical predictors of metastasis.

In summary, our results demonstrate the use of clinical and genomic predictors to improve personalized decision-making following RP. These tools may encourage some men and their providers to select observation following prostatectomy despite the presence of adverse pathological features and a detectable PSA. For men at high clinico-genomic risk, adjuvant radiation therapy may be selected as it results in the lowest incidence of metastatic disease. Indeed, for men at highest risk, clinical trials incorporating novel agents on a backbone of adjuvant radiation therapy should be encouraged. Ultimately, these results could support an individualized approach to the management of men with adverse pathological features following prostatectomy. This would have major ramifications for patient quality of life and the efficient use of health-care resources.

## Figures and Tables

**Table 1:** Demographic and clinical characteristics of eligible patients (*n*=422)

*Variables*	*ART*	*MRD-SRT*	*SRT*	*No RT*	P^a^
No. patients (%)	111	70	83	158	
Patient age, year					0.85
Median (Q1, Q3)	60 (57, 64.5)	60.5 (56.2, 64)	62 (56.5, 66)	62 (57, 65)	
Preoperative PSA, ng ml^−1^					0.07
Median (Q1, Q3)	7.2 (5.3, 10.6)	7.7 (5.4, 12.3)	8.3 (5.2, 15.5)	8.8 (5.8, 13)	
Pathological Gleason score, *n* (%)					0.13
⩽3+4	59 (53.2)	50 (71.4)	41 (49.4)	84 (53.2)	
4+3	24 (21.6)	13 (18.6)	24 (28.9)	32 (20.3)	
8	15 (13.5)	4 (5.7)	9 (10.8)	18 (11.4)	
⩾9	13 (11.7)	3 (4.3)	9 (10.8)	24 (15.2)	
Extraprostatic extension, *n* (%)					<0.001
	65 (58.6)	34 (48.6)	50 (60.2)	118 (74.7)	
Seminal vesicle invasion, *n* (%)					0.23
	36 (32.4)	15 (21.4)	24 (28.9)	36 (22.8)	
Positive surgical margins, *n* (%)					<0.001
	92 (82.9)	59 (84.3)	71 (85.5)	76 (48.1)	
Concurrent ADT, *n* (%)					<0.001
	10 (9.1)	9 (12.9)	15 (18.1)	0 (0.0)	
Time from RP to RT, months					NA
Median (Q1, Q3)	5 (3, 10)	13 (6, 29)	9 (5, 31)	NA	
PSA at RT inititation, ng ml^−1^					NA
Median (Q1, Q3)	0.10 (0.05, 0.19)	0.30 (0.25, 0.40)	1.00 (0.65, 2.20)	NA	
Metastasized, *n*					NA
	3	4	11	19	

Abbreviations: ADT, androgen deprivation therapy; ART, adjuvant radiation therapy; MRD, minimal residual disease; RP, radical prostatectomy; SRT, salvage radiation treatment

.

a *P*-values are computed using analysis of variance F-test if the variable is continuous or Fisher's exact test if the variable is categorical. All *P*-values are two-sided.

**Table 2:** Cox multivariable analysis of treatment groups adjusted by Decipher and CAPRA-S

	*Risk factor*	*Hazard ratio (95% CI)*	P
Panel A—CAPRA-S continuous	Decipher^a^	1.28 (1.08–1.52)	0.004
	CAPRA-S^b^	1.39 (1.18–1.62)	<0.001
	ART	Reference	1
	MRD-SRT	2.30 (0.51–10.33)	0.28
	SRT	4.31 (1.20–15.47)	0.02
	No RT	5.42 (1.59–18.44)	0.007
Panel B—CAPRA-S categorical	Decipher^a^	1.26 (1.05–1.50)	0.01
	CAPRA-S⩽5	Reference	1
	CAPRA-S 6-12	5.37 (2.48–11.65)	<0.001
	ART	Reference	1
	MRD-SRT	2.51 (0.56–11.31)	0.23
	SRT	4.52 (1.26–16.21)	0.02
	No RT	5.31 (1.57–18.03)	0.007

Abbreviations: ART, adjuvant radiation treatment; CAPRA-S, cancer of the prostate-risk assessment post-surgical; CI, confidence interval; MRD, minimal residual disease; SRT, salvage radiation treatment.

a Decipher reported per 10% increase.

b CAPRA-S reported per unit increase.
