# Supplementary material for: Efficacy of post-operative radiation in a prostatectomy cohort adjusted for clinical and genomic risk
Source: Prostate Cancer Prostatic Dis. 2016 May 3;19(3):277–82. doi: 10.1038/pcan.2016.15 (PMC5411670; doi:10.1038/pcan.2016.15)

**Table S1.** Cox multivariable analysis of treatment groups adjusted by Decipher or CAPRA-S.

**Table S2.** Cox multivariable analysis of treatment groups adjusted by Decipher, CAPRA-S and concurrent ADT.

**Table S3.** Cox multivariable analysis of treatment groups adjusted by Decipher, CAPRA-S. MRD-SRT and SRT were treated as time-dependent covariates.

**Table S4.** Cox multivariable analysis of treatment groups adjusted by Decipher, and CAPRA-S excluding patients who received ADT after RT (n=414).

**Table S5.** Predicted 10-year risk of metastasis according to CAPRA-S and Decipher risk groups for each treatment arm (n=422).

**Figure S1.** Distribution of patients by **(A)** CAPRA-S and **(B)** Decipher. Median CAPRA-S score for men who underwent ART, MRD-SRT, SRT, and no RT was 5 (IQR, 4-6), 5 (IQR, 4-6), 4 (IQR, 4-6), and 4 (IQR, 4-6), respectively. Median Decipher scores for men who received ART, MRD-SRT, SRT, and no RT was 0.42 (IQR, 0.28-0.55), 0.46 (IQR, 0.27-0.59), 0.41 (IQR, 0.28-0.57), and 0.34 (IQR, 0.21-0.52), respectively.

**Figure S2.** Prediction curves of metastasis for treatment groups at 10 years adjusted by (A) CAPRA-S and (B) Decipher score.

**Figure S3.** Prediction curves of metastasis for treatment groups at 10 years adjusted by categorical CAPRA-S and Decipher score. Adjusted ART rates are calculated based on the formula described by King^26^ using the 10-year BCR-free survival from the RP alone arm of SWOG 8794^3^.

**Table S1.**

|  | **CAPRA-S Score**** | | **Decipher Score***** | |
| --- | --- | --- | --- | --- |
| **Variable** | **Hazard Ratio (95% CI)** | **P** | **Hazard Ratio (95% CI)** | **P** |
|  |  |  |  |  |
| Age | 1 (0.95-1.05) | 0.94 | 0.99 (0.94-1.04) | 0.63 |
| Risk Model* | 1.44 (1.24-1.66) | <0.001 | 1.38 (1.17-1.63) | <0.001 |
| ART | ref | 1 | ref | 1 |
| MRD-SRT | 2.35 (0.52-10.53) | 0.26 | 1.74 (0.39-7.82) | 0.47 |
| SRT | 4.11 (1.14-14.77) | 0.03 | 4.51 (1.25-16.24) | 0.02 |
| No RT | 4.76 (1.40-16.14) | 0.01 | 4.57 (1.35-15.52) | 0.01 |
| Abbreviations: ART, adjuvant radiation treatment; CAPRA-S, cancer of the prostate risk assessment post-surgical score | | |  |  |
| CI, confidence interval; MRD, minimal residual disease; SRT, salvage radiation treatment | | | |  |
| *Risk model refers to Decipher or CAPRA-S |  |  |  |  |
| **CAPRA-S reported per unit increase |  |  |  |  |
| ***Decipher reported per 10% increase |  |  |  |  |

**Table S2.**

| **Risk Factor** | **Hazard Ratio (95% CI)** | **P** |
| --- | --- | --- |
|  |  |  |
| Decipher* | 1.28 (1.08-1.52) | 0.002 |
| CAPRA-S** | 1.39 (1.18-1.62) | <0.001 |
| ART | ref | 1 |
| MRD-SRT | 2.28 (0.51-10.27) | 0.28 |
| SRT | 4.28 (1.19-15.41) | 0.03 |
| No RT | 5.38 (1.54-18.84) | 0.008 |
| Concurrent ADT | 1.00 (0.29-3.46) | 1 |
| Abbreviations: ADT, androgen deprivation therapy ART, adjuvant radiation treatment | | |
| CAPRA-S, cancer of the prostate risk assessment post-surgical score; CI, confidence interval | | |
| MRD, minimal residual disease; SRT, salvage radiation treatment | |  |
| *Decipher reported per 10% increase |  |  |
| **CAPRA-S reported per unit increase |  |  |

**Table S3.**

| **Risk Factor** | **Hazard Ratio (95% CI)** | **P** |
| --- | --- | --- |
|  |  |  |
| Decipher* | 1.27 (1.07-1.51) | 0.006 |
| CAPRA-S** | 1.37 (1.18-1.61) | <0.001 |
| ART | ref | 1 |
| MRD-SRT | 2.73 (0.61-12.28) | 0.19 |
| SRT | 5.34 (1.49-19.21) | 0.01 |
| No RT | 4.40 (1.29-14.97) | 0.02 |
| Abbreviations: ADT, androgen deprivation therapy ART, adjuvant radiation treatment | | |
| CAPRA-S, cancer of the prostate risk assessment post-surgical score; CI, confidence interval; SRT, salvage radiation treatment | | |
| MRD, minimal residual disease; SRT, salvage radiation treatment | |  |
| *Decipher reported per 10% increase |  |  |
| **CAPRA-S reported per unit increase |  |  |

**Table S4.**

| **Risk Factor** | **Hazard Ratio (95% CI)** | **P** |
| --- | --- | --- |
|  |  |  |
| Decipher* | 1.25 (1.05-1.49) | 0.01 |
| CAPRA-S** | 1.42 (1.21-1.66) | <0.001 |
| ART | ref | 1 |
| MRD-SRT | 2.48 (0.55-11.16) | 0.23 |
| SRT | 5.20 (1.45-18.67) | 0.01 |
| No RT | 5.46 (1.60-18.58) | 0.007 |
| Abbreviations: ADT, androgen deprivation therapy ART, adjuvant radiation treatment | | |
| CAPRA-S, cancer of the prostate risk assessment post-surgical score; CI, confidence interval; SRT, salvage radiation treatment | | |
| MRD, minimal residual disease; SRT, salvage radiation treatment | |  |
| *Decipher reported per 10% increase |  |  |
| **CAPRA-S reported per unit increase |  |  |

**Table S5.**

| **10-year risk of metastasis (95% CI)** | | | | |
| --- | --- | --- | --- | --- |
| **Panel A - CAPRA-S Score** | **ART** | **MRD-SRT** | **SRT** | **No RT** |
| Low and Intermediate (0-5) | 1% (0-3%) | 3% (0-7%) | 6% (1-10%) | 6% (2-10%) |
| High (6-12) | 8% (0-17%) | 20% (0-36%) | 32% (13-47%) | 34% (18-47%) |
| **10-year risk of metastasis (95% CI)** | | | | |
| **Panel B - Decipher Score** | **ART** | **MRD-SRT** | **SRT** | **No RT** |
| Low (<0.45) | 2% (0-5%) | 4% (0-9%) | 10% (2-16%) | 10% (4-16%) |
| Intermediate (0.45-0.60) | 4% (0-8%) | 6% (0-13%) | 14% (2-24%) | 15% (3-25%) |
| High (>0.60) | 11% (0-23%) | 19% (0-35%) | 38% (13-56%) | 40% (16-57%) |
| Abbreviations: ART, adjuvant radiation treatment | |  |  |  |
| CI, confidence interval; MRD, minimal residual disease; SRT, salvage radiation treatment | | | |  |

**Figure S1.**
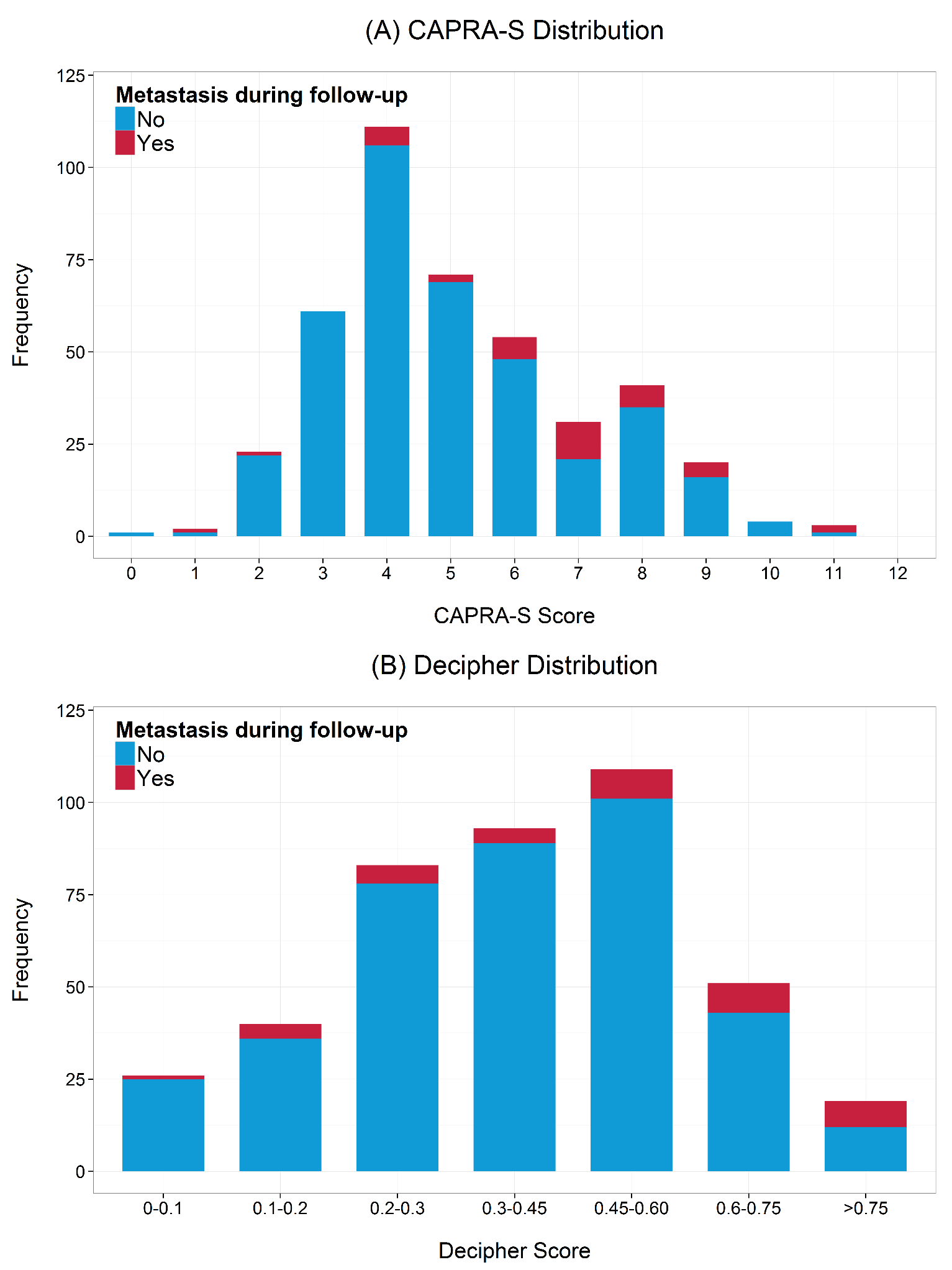


**Figure S2.
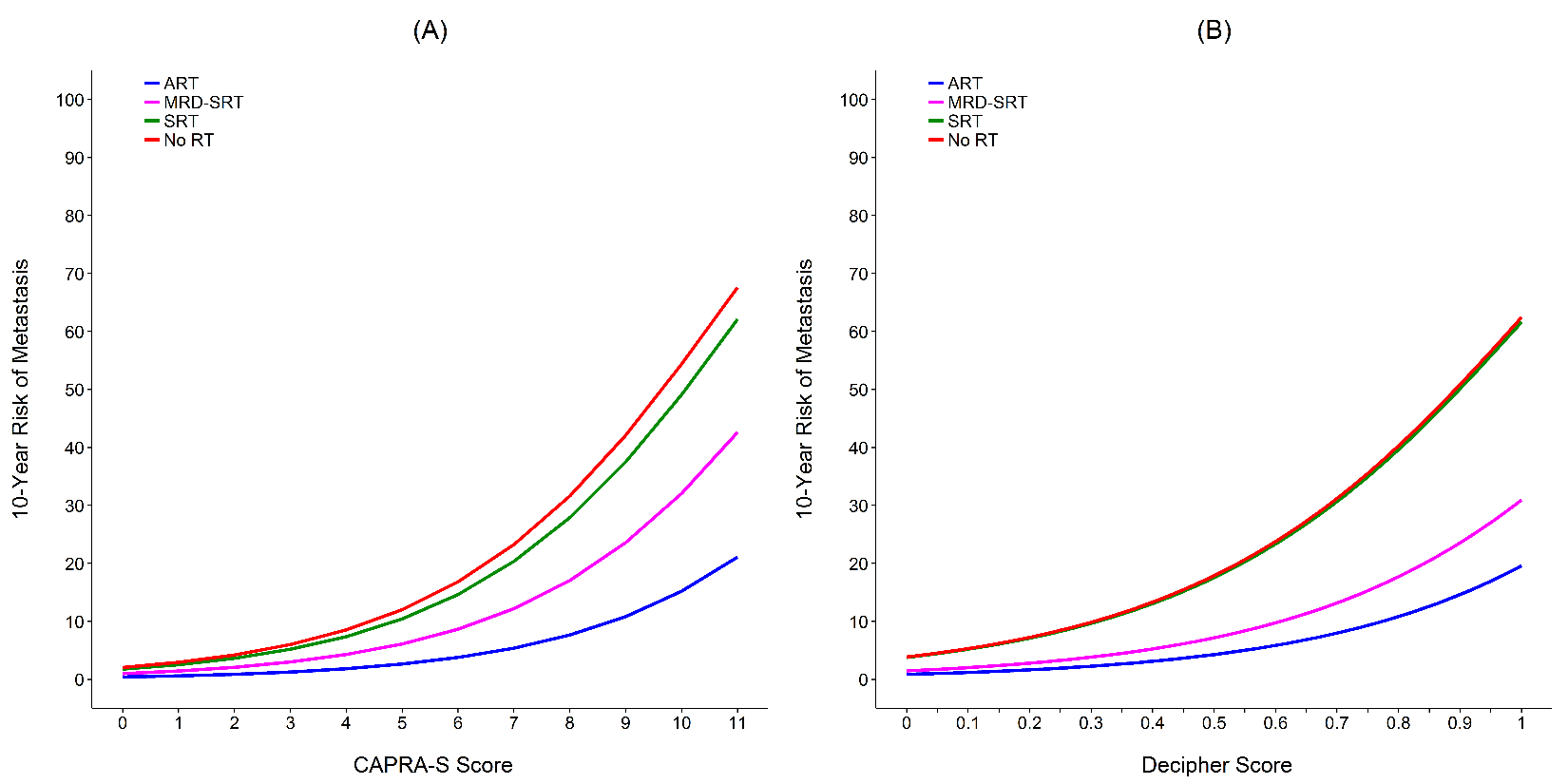
**

**Figure S3.**


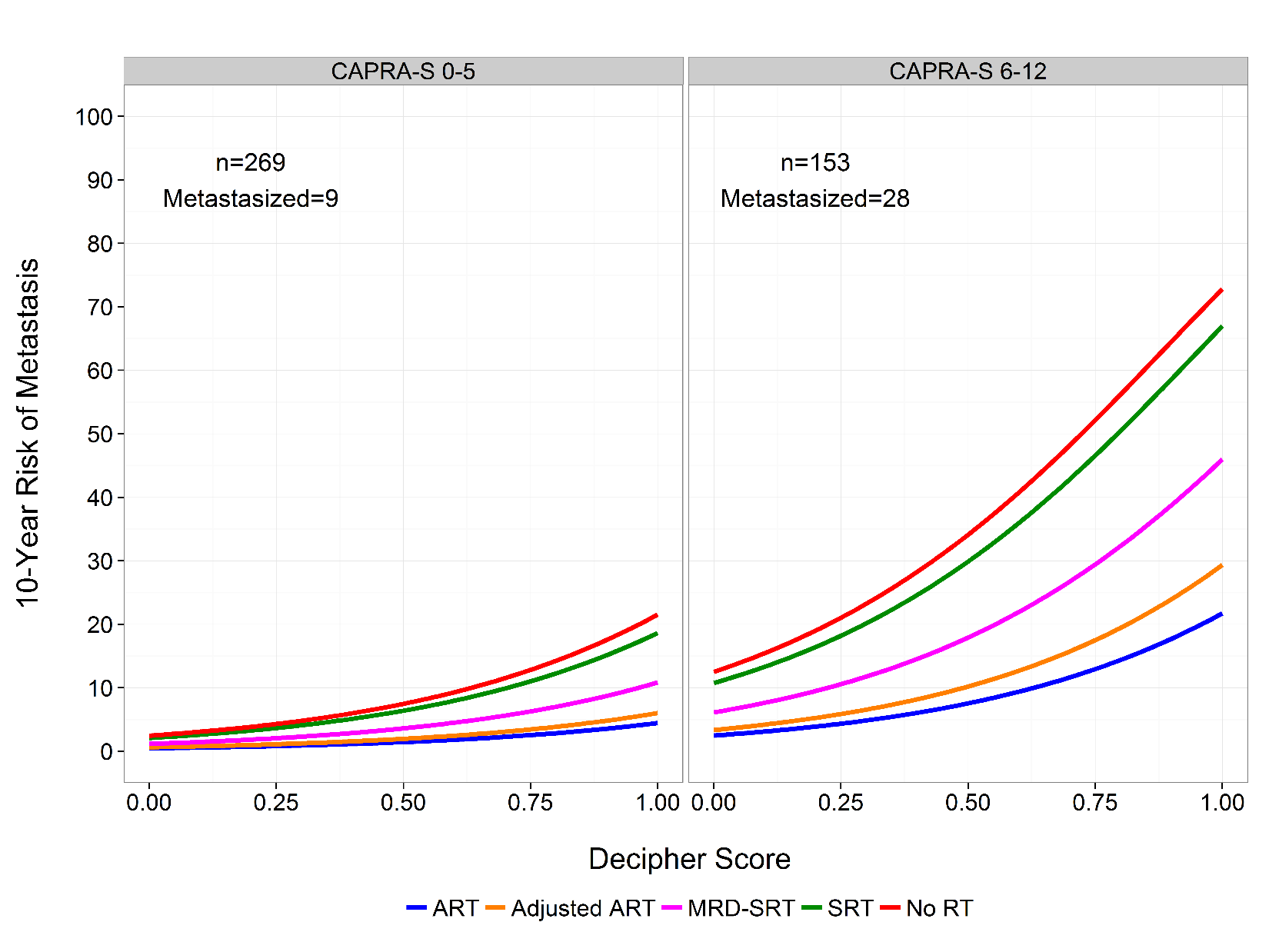

Supplement: Supplementary Information [file pcan201615x1.docx]
